# C.I. Basic Red 46 Removal from Sewage by Carbon and Silica Based Composite: Equilibrium, Kinetic and Electrokinetic Studies

**DOI:** 10.3390/molecules27031043

**Published:** 2022-02-03

**Authors:** Małgorzata Wiśniewska, Stanisław Chibowski, Monika Wawrzkiewicz, Magda Onyszko, Viktor Bogatyrov

**Affiliations:** 1 Department of Radiochemistry and Environmental Chemistry, Faculty of Chemistry, Institute of Chemical Sciences, Maria Curie-Sklodowska University in Lublin, M. Curie-Sklodowska Sq. 3, 20-031 Lublin, Poland; stanislaw.chibowski@poczta.umcs.lublin.pl; 2 Department of Inorganic Chemistry, Faculty of Chemistry, Institute of Chemical Sciences, Maria Curie-Sklodowska University in Lublin, M. Curie-Sklodowska Sq. 2, 20-031 Lublin, Poland; monika.wawrzkiewicz@mail.umcs.pl (M.W.); magda.onyszko97@gmail.com (M.O.); 3 Chuiko Institute of Surface Chemistry, National Academy of Sciences of Ukraine, General Naumov Street 17, 03164 Kyiv, Ukraine; bogatyrov@isc.gov.ua

**Keywords:** basic dye removal, adsorption, composite, textile wastewaters, zeta potential, dye–surfactant complexes

## Abstract

The worldwide production of colored products and intermediates is increasing year on year. The consequence of this is an increase in the number of liquid effluents containing toxic dyes entering the aquatic environment. Therefore, it is extremely important to dispose of them. One of the techniques for the elimination of environmentally harmful dyes is adsorption. The main purpose of this study was to explore the possibility of using a carbon and silica (C/SiO_2_)-based composite for the removal of the azo dye C.I. Basic Red 46 (BR46). The adsorption capacity of C/SiO_2_ was found to be temperature dependent and increased from 41.90 mg/g to 176.10 mg/g with a temperature rise from 293 K to 333 K in accordance with the endothermic process. The Langmuir isotherm model seems to be the better one for the description of experimental data rather than Freundlich or Dubinin–Radushkevich. The free energy (ΔG^o^) confirmed the spontaneous nature of BR46 adsorption by C/SiO_2_. Kinetic parameters revealed that BR46 uptake followed the pseudo-second-order equation; however, the external diffusion plays a significant role. Surfactants of cationic, anionic and non-ionic type influenced BR46 retention by C/SiO_2_. The electrokinetic results (solid surface charge density and zeta potential) indicated that the adsorption of cationic dye and surfactant influences the structure of the electrical double layer formed at the solid–liquid interface.

## 1. Introduction

Dyes are organic substances that are used in many areas of industry such as: textiles, paper, tanning, cosmetics and food industries. Today, more than 100,000 dyes are known and available in the market, and their annual production reaches even 70,000 tons [1,2]. The presence of synthetic dyes in effluents discharged into the aquatic environment contributes to changes in the physicochemical parameters of surface water, reduces the permeability of sunlight and thus inhibits the process of photosynthesis. In addition, there are dyes which in their structure contain heavy metals such as chromium, nickel and copper (i.e., metal complex dyes), and therefore have carcinogenic and mutagenic properties. Basic dyes have significant toxicity compared to other classes of dyes [3]. Many dyes do not undergo biological decomposition, which makes them a great threat to the environment, as well as to human health and life [1,2]. Therefore, it is becoming increasingly important to find an effective and economical way of removing dyes from the industrial effluents. There is a lot of information available in the literature about the methods of their removal [4,5,6,7,8]. These methods can be divided into chemical and physical ones. Chemical methods include photocatalytic degradation, flocculation, coagulation and oxidation. Physical methods include membrane filtration and adsorption. Among the abovementioned techniques for the removal of dyes from wastewater, adsorption is very popular. This is a simple technique in terms of technology and is very efficient and economical, because it allows the use of sorbents of both natural and synthetic origin, as well as industrial waste materials [1,2,3,4,5,8]. At present, the most popular adsorbents are materials based on activated carbon due to its developed specific surface area and high porosity, as well as high efficiency in the removal of dyes from wastewater [9]. They possess a number of favorable properties, among which are resistance to high temperatures in non-oxidizing atmospheres, chemical stability, resistance to drastic temperature changes, or low coefficients of thermal expansion [10]. Even more promising are mixed adsorbents representing groups of carbon composites with inorganic oxides, such as SiO_2_, Al_2_O_3_, Fe_2_O_3_ or TiO_2_. The forms of carbon in these materials can be different, and among others, they can be carbon nanotubes, activated carbon, biochar, flay ash, graphene and its derivatives. Such solids are widely used in the removal process of undesirable or toxic substances from the aqueous phase. Iron oxide (Fe_3_O_4_)-activated carbon obtained from peanut shell was applied successfully in Cr(VI) ion removal [11]. Silica/activated carbon composite turned out to be very effective for Ni(II) adsorption from aqueous solution in comparison to the precursors such as silica and activated carbon [12]. Carbon nanotubes covered with magnetic Fe/Zn-layered double oxide were successfully used in U(VI) and ^241^Am(III) removal [13]. Zero-valent iron particles after their coverage with starch and immobilization on an activated carbon surface were applied in Cr(VI) ion separation [14]. Moreover, zero-valent iron deposited on biochar carrier was used in the Cd(III) and As(III) co-adsorption process in aqueous solution [15]. Very effective adsorbent composed of the multi-walled carbon nanotubes and alumina for simultaneous Cd(II) and trichloroethylene removal from groundwater was elaborated upon [16]. On the other hand, magnetic graphene oxide was synthesized and used in the simultaneous elimination of Cd(II) and ionic dyes, such as methylene blue and orange G, from real water samples [17]. It was also shown that carbon–silica composite presents high efficiency for Cu(II) adsorption from a solution containing proteins with a different internal stability (ovalbumin and lysozyme) [18] as well as ionic polyacrylamides [19]. Waste material such as fly ash (consisting of unburned carbon, silica, alumina and iron oxide) was used for the separation of copper, nickel and cadmium ions in the presence of methylene blue dye [20].

One of the main pollutants of the aquatic environment are surfactants, used intensively both in households as washing and cleaning agents, and in many industries. Their negative effect is primarily due to their indirect harmfulness to aquatic ecosystems. Surfactants facilitate the dissolution of toxic substances, which are sparingly soluble or insoluble in water. They also cause water foaming, worsening the conditions of oxygen diffusion. The consequences of this are the decline of biological life and the deterioration of the self-cleaning capacity of water reservoirs. Additionally, literature reports indicate that nonionic surfactants may be directly toxic, stronger than ionic ones [21,22]. The effects of surfactants with different ionic character on the dye adsorption were examined in our previous papers. It was proved that surface active substances affect the adsorption of dyes, influencing their removal from aqueous solutions [9,23,24]. For this reason, it is important to know their impact on the mechanism of removal of other undesirable and toxic substances, which may contribute to the development of more effective methods of their disposal.

This paper describes the adsorption properties of the carbon-and-silica-based composite towards basic dye. The mechanism of dye retention through the composite was evaluated based on popular adsorption isotherm models and equilibrium experiments. Moreover, the kinetic parameters of dye sorption were calculated, and the effects of the presence of cationic, anionic and non-ionic surfactants on the sorption capacity of the composite were evaluated. The sign and charge magnitude of adsorbent/adsorbate interface were determined using potentiometric titration and Doppler lase electrophoresis methods. They enabled the determination of the surface charge density and zeta potential of the solid particles in the systems without and with cationic dye and surfactants of different ionic character.

## 2. Results

### 2.1. Effect of pH

**Figure 1:**
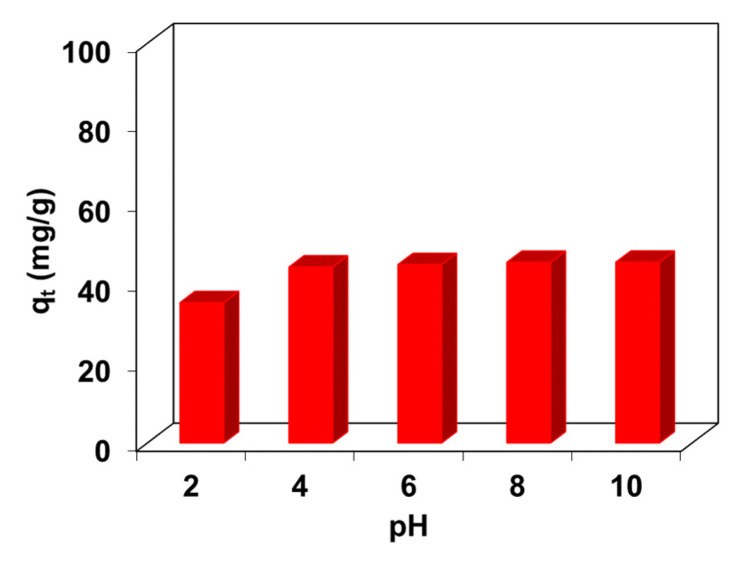
Effect of solution pH on the BR46 uptake from the solution of the initial concentration 100 mg/L.

pH is an important parameter affecting the adsorption efficiency of dyes. The effect of pH on the adsorption of BR46 by the C/SiO_2_ composite was investigated in the pH range from 2 to 10 and is shown in Figure 1. The amount of BR46 adsorbed (q_t_) by C/SiO_2_ after sorption time t was calculated from Equation (1):

(1) $$q_{t}=\frac{\left(C_{0}-C_{t}\right)}{m}V$$

where *C*_0_—BR46 initial concentration (mg/L), *C_t_*—BR46 concentration after sorption time *t* (mg/L), *V*—solution volume (L) and *m*—adsorbent mass (g).

It increased with increasing pH and showed a maximum value in the 4–10 range. The lower uptake of BR46 in strongly acidic media is due to the presence of excess H^+^ ions which compete with cationic dye for adsorption sites. Similar observations were described during BR46 uptake by the biochar prepared from *Chrysanthemum morifolium* Ramat straw [1], Algerian natural phosphates [25], gypsum [26] and Moroccan clay [27].

### 2.2. Equilibrium Experiments

The Freundlich, Langmuir and Dubinin–Radushkevich adsorption isotherms [1,2,28] were proposed as helpful tools for the determination of the interaction between the BR46 and C/SiO_2_ in order to explain the equilibrium states of the adsorption process. The abovementioned isotherm models are described using the following linear Equations (2)–(4) [1,2,28]:

(2) $$\log q_{e}=logk_{F}+\frac{1}{n}logC_{e}$$

(3) $$\frac{C_{e}}{q_{e}}=\frac{1}{Q_{0}k_{L}}+\frac{C_{e}}{Q_{0}}$$

(4) $$lnq_{e}=lnq_{m}-k_{DR}\varepsilon^{2}$$

where *q_e_*—adsorption capacity (mg/g), *k_F_*—the Freundlich constant (mg^1−1/n^∙L^1/n^/g), 1/*n* –parameter characterizing the energy heterogeneity of the adsorbent surface, *C_e_*—BR46 concentration at equilibrium (mg/L), *Q_0_*—monolayer capacity (mg/g), *k_L_*—the Langmuir constant (L/mg), *q_m_*—maximum adsorption capacity (mg/g), *k_DR_*—constant related to the adsorption energy (mol^2^/J^2^) and *ε*—adsorption potential (J/mol) (calculated as $\varepsilon =RTln\left(1+\frac{1}{C_{e}}\right)$, where *R*—gas constant 8.314 J/mol·K and *T*—temperature (K)).

In the BR46-C/SiO_2_ system, the Freundlich isotherm assumes multilayer adsorption of BR46 species on a heterogeneous composite surface. The Langmuir isotherm is based on the assumption that BR46 molecules form a monomolecular layer on specific and uniform sites on the composite, possessing equal energy [1,2]. The adsorbed molecules of BR46 are immobile, and do not interact. On other hand, the Dubinin–Radushkevich isotherm is often used to estimate the apparent free energy of adsorption as well as differentiate between the physical and chemical adsorption mechanism based on Equation (5) [28]:

(5) $$E=\frac{1}{\sqrt{2k_{DR}}}$$

where *E*—mean free energy for BR46 removal from its adsorption site to the infinity (J/mol).

As reported in [1], if the E is between 1 and 8 kJ/mol, the adsorbate uptake is the result of physical interactions, while if it is between 8 and 16 kJ/mol, it depends on the ion exchange, and E values higher than 16 kJ/mol suggest chemical adsorption.

By applying linear regression to the obtained experimental data in the BR46-C/SiO_2_ system, the parameters of isotherms were calculated and are presented in Table 1.

**Figure 2:**
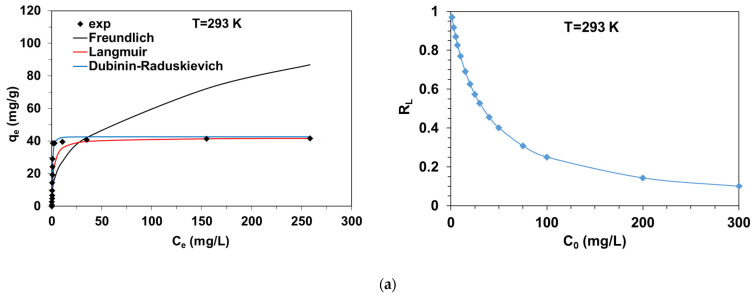

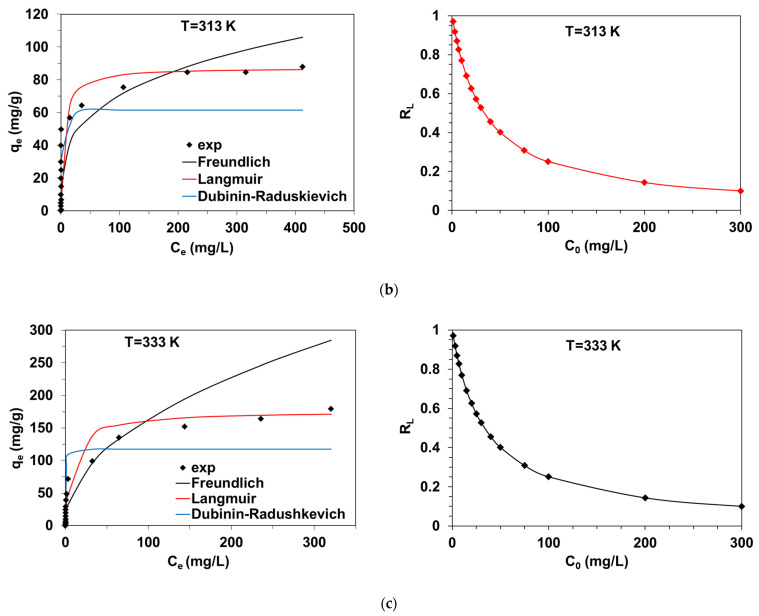
Comparison of the experimental adsorption data of BR46 on C/SiO_2_ with fitting curves corresponding to the Freundlich, Langmuir and Dubinin–Radushkevich as well as changes of the separation factor (*R_L_*) at (**a**) 293 K, (**b**) 313 K and (**c**) 333 K.

A thorough analysis of the parameters of isotherms determined by the linear regression method allows us to conclude that the Langmuir model can be applied to describe the dynamic equilibrium of the BR46-C/SiO_2_ system. Values of the determination coefficients *R*^2^ equaled 0.999 at 293 K, 0.996 at 313 K and 0.990 at 333 K. These values were higher than those determined for the Freundlich (*R*^2^ = 0.470–0.739) or Dubinin–Radushkevich (*R*^2^ = 0.608–0.986) models. In addition, the smallest values of the residual sum of squares error (ERRSQ/SSE), Chi-square (χ^2^) and sum of squares regression (SSR) were obtained for the Langmuir model. Figure 2 shows the fitting curves of the isotherm models to the experimental data. The plateau corresponding to the monolayer capacities is observed. The Q_0_ enlarged from 41.90 mg/g to 176.10 mg/g with a temperature rise from 293 K to 333 K in agreement with the endothermic process. The fact that the dye uptake was favored with a temperature rise indicates that the mobility of BR46 molecules increased. The high temperature favored BR46 molecules’ diffusion in the internal porous structure of the composite and increased interaction between sorbate cations and functional groups of the adsorbent. The values of the dimensionless equilibrium parameter R_L_ [25] (defined as $R_{L}=\frac{1}{1+k_{L}C_{0}}$), being essential characteristics of the Langmuir model, were found to be in the 0–1 range, which means the favorable adsorption of BR46 on C/SiO_2_.

The apparent free energies of adsorption E calculated based on the Dubinin–Radushkevich isotherm model, included in Table 1, are in the range of 1.66–2.73 kJ/mol. Moreover, the calculated q_m_ values, especially at temperatures of 313 K and 333 K, deviate from the experimental values, as shown in Figure 2. In view of the above, the Dubinin–Radushkevich model cannot be used to describe the system under study due to the lower values of *R*^2^ being in the range of 0.608–0.986.

Comparison of the adsorption capacities of the C/SiO_2_ composite with those of other adsorbents [25,26,27,28,29,30,31,32] used for BR46 removal allows us to conclude that it can be considered as an adsorptive material on an industrial scale in a wastewater treatment plant containing BR46 (Table 2).

Thermodynamic parameters such as free energy (Δ*G*^o^), standard enthalpy (Δ*H*^o^) and entropy (Δ*S*^o^) were calculated using Equations (6)–(7) [30,33]:

(6) $$\Delta G^{o}=-RTlnK_{c}c^{0}\;$$

(7) $$lnK_{c}c^{0}=\frac{\Delta S^{o}}{R}-\frac{\Delta H^{o}}{RT}\;$$

where *R*—gaseous constant (8.314 J/mol K), *T*—temperature (K), *K_c_*—distribution constant at equilibrium calculated as $K_{c}=\frac{q_{e}}{C_{e}}\;$(L/g) and *c*^0^—concentration of the standard reference solution (*c^0^* ≡ 1000 g/L [33]).

**Figure 3:**
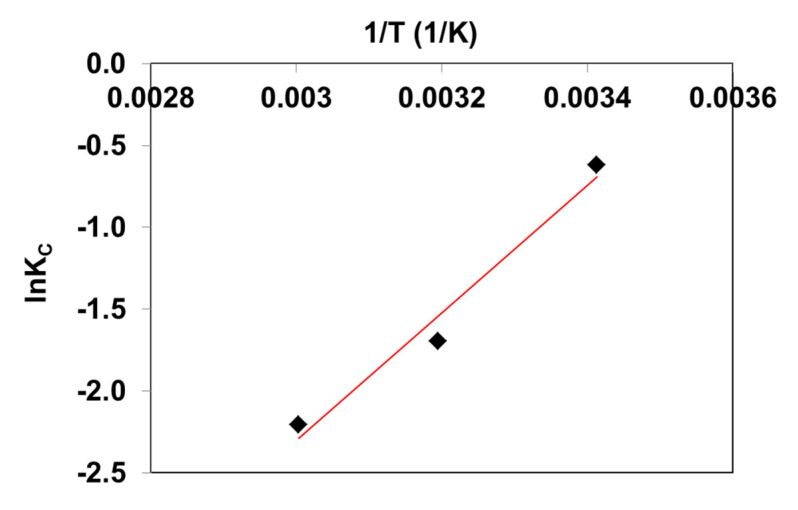
1/*T* dependency of ln*K_C_* for BR46 uptake by C/SiO_2_ composite.

The determination of the slope and intercept of the linear plot *lnK_C_* vs. 1/*T* (Figure 3) enables the calculation of standard enthalpy and entropy.

The negative values of free energy were found to be −15 kJ/mol, −14 kJ/mol and −13 kJ/mol at 293 K, 313 K and 333 K, respectively, suggesting that the BR46 process is spontaneous. Δ*G*^o^ values were in the range of 0 to −20 kJ/mol, which indicated that BR46 adsorption on the composite is the result of a physical adsorption process [31]. Similar values of ΔG^o^ (between −14 kJ/mol and −13 kJ/mol) were reported by Olgun and Atar [30], who studied BR46 adsorption in boron industry waste. The spontaneous nature of BR46 adsorption on activated carbon obtained from palm bio-waste was confirmed by Kiani Ghaleh Sardi et al. [31]. The standard enthalpy was found to be −32 kJ/mol for BR46 interactions with C/SiO_2_ composite. In addition, negative values of Δ*S*^o^ (−116 J/mol) indicate a decrease in disordernation upon the adsorption of BR46 on C/SiO_2_. Entropy values at a similar level (i.e., −121 J/mol) were determined in the case of BR46 adsorption on boron-waste-based adsorbent [30].

### 2.3. Kinetic Experiments

The next step of the experiment was to establish the impact of contact time on the dye uptake by C/SiO_2_ composite. The BR46 removal was studied from the solutions of 25 mg/L, 50 mg/L and 100 mg/L initial concentrations at predetermined time intervals ranging from 1 to 240 min. The kinetic parameters of sorption were calculated from the linear forms of the pseudo-first order (PFO), pseudo-second order (PSO) and intraparticle diffusion (ID) using Equations (8)–(10) [34,35,36,37,38,39]:

(8) $$\log(q_{e}-q_{t})=log\left(q_{t}\right)-\frac{k_{1}}{2.303}t$$

(9) $$\frac{t}{q_{t}}=\frac{1}{k_{2}q_{e}^{2}}+\frac{1}{q_{e}}t_{\;}$$

(10) $$q_{t}=k_{i}t^{0.5}$$

where *q_e_*—adsorption capacity (mg/g), *q_t_* (mg/g)—amount of BR46 adsorbed at time *t* (min) per unit mass of composite and *k*_1_ (1/min), *k*_2_ (g/mg min) and *k_i_* (mg/g min^0.5^)—rate constants of sorption calculated from the PFO, PSO and ID equations, respectively.

**Figure 4:**
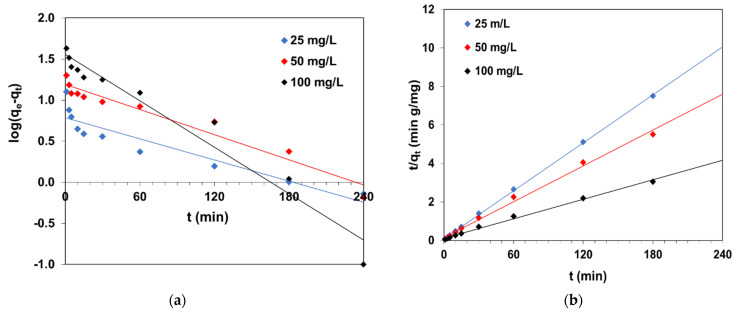

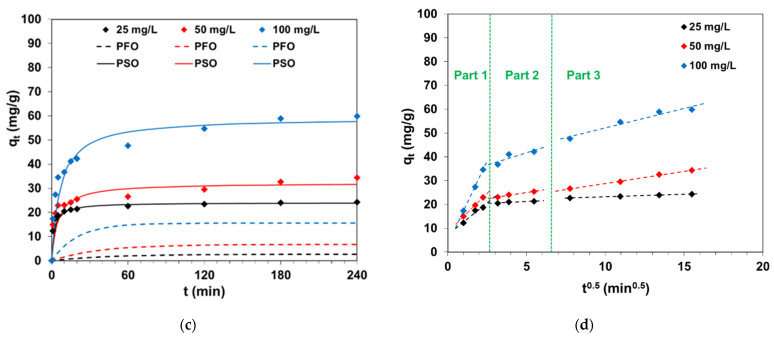
(**a**) PFO and (**b**) PSO kinetic plots, (**c**) fitting curves of experimental data to PFO and PSO models and (**d**) intraparticle diffusion graph in the BR46–C/SiO_2_ system.

Figure 4 presents kinetic plots for the PFO model and PSO model, fitting of the experimental points to PFO and PSO equations as well as an intraparticle diffusion plot for BR46 sorption by C/SiO_2_ composite. An increase in contact time increased the chances of interactions between the C/SiO_2_ and dye cations; however, after a particular period, the adsorption rate became constant due to equilibrium attainment between phases. The higher the BR46 concentration in the solution, the longer time it took to achieve the state of equilibrium. It was found that 60 min was sufficient to reach equilibrium in solutions with an initial dye concentration of 25 mg/L. It was observed that the dynamic equilibrium occurred after 180 min in the solutions with the initial dye concentrations of 50 mg/L and 100 mg/L.

One can see that BR46 adsorption on C/SiO_2_ obeys the pseudo-second-order model. This is confirmed by the linear plot *t*/*q_t_* vs. *t* and the largest determination coefficients *R*^2^ being in range of 0.996–0.999 depending on the dye concentration. The *q_e_* values calculated on the basis of the PSO model (24.1 mg/g, 32.2 mg/g and 59.2 mg/g for the solutions containing 25 mg/L, 50 mg/L and 100 mg/L of BR46, respectively) are similar to the values determined experimentally *q_e,exp_* (25.0 mg/g, 34.5 mg/g and 60.0 mg/g), which, apart from the values of the determination coefficients *R*^2^ (Table 3), confirmed its use in the kinetic description of the tested systems. The rate constants *k*_2_ drop with increasing BR46 content in the solution. It is believed that at higher concentrations, the initial adsorption leads to swelling of the adsorbent, thus exposing the inner surface and pores for greater adsorption. This part of the process is likely to be slower due to diffusion of this large molecule to this hidden surface in the pores of the adsorbent [35].

Fitting lines corresponding to the PFO model deviate significantly from the experimental data, as shown in Figure 4a. The values of the kinetic parameters of PFO calculated from the plot *log(q_e_ − q_t_)* vs. *t* and listed in Table 3 indicate that it cannot be used to describe the experimental data in the studied system.

In order to identify the rate-controlling step of adsorption, the intraparticle diffusion model was considered. Figure 4d revealed multilinearity of the graph *q_t_* vs. *t*^0.5^ with three distinct regions: external diffusion (Part 1), intraparticle diffusion (Part 2) and equilibrium adsorption (Part 3) [39]. The initial part relates to BR46 diffusion through the thin layer surrounding the adsorbent beads. The values of the determination coefficients (*R*^2^_1_) are equal to 0.953, 0.999 and 0.999 and indicate that this stage can be considered as one of the rate-limiting steps in the adsorption process. An increase in *k_i_*_1_ was observed with increasing BR46 concentration (Table 3). The second part of the graph reflects intraparticle diffusion (*R*^2^_2_ ranged from 0.725 to 0.978), while the third part describes equilibrium (the plateau was observed, *R*^2^_3_ = 0.953–0.995).

**Figure 5:**
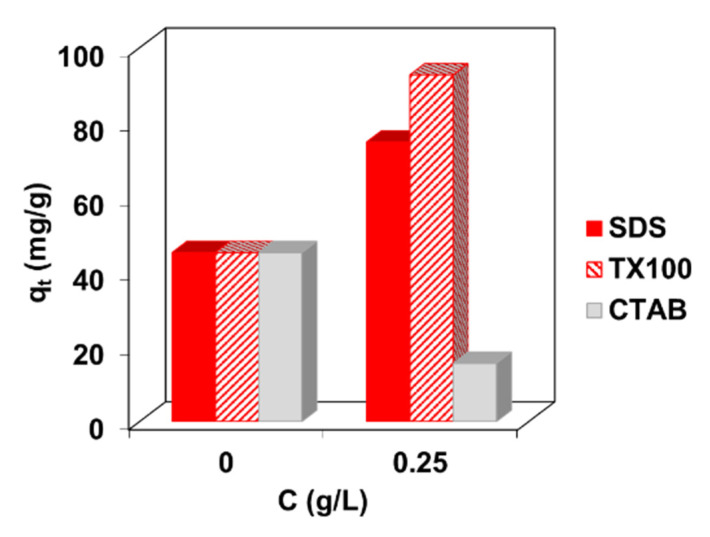
Influence of the surfactants presence on BR46 uptake by C/SiO_2_ in the 100 mg/L BR46 + 0.25 g/L surfactants (SDS, TX100, CTAB) systems.

The next stage of the research was to evaluate the influence of surfactants on the dye adsorption efficiency on the composite. For this purpose, the batch adsorption was carried out in the presence of the anionic, cationic and non-ionic surfactants such as SDS, TX100 and CTAB from the solutions containing 100 mg/L of BR46 and 0.25 g/L of each surfactant (Figure 5).

It can be seen in Figure 5 that anionic surfactant SDS and non-ionic TX100 enlarged BR46 uptake by the composite which is due to enhanced adsorption of the dye cations as well as its aggregates with negatively charged SDS or complexes with nonionic TX100. The opposite effect was observed in systems containing 0.25 g/L CTAB, where there was a decrease in dye adsorption compared to systems not containing this surfactant. This is the result of competitive adsorption of CTAB compared to the cationic form of the dye on the negatively charged adsorbent surface. The evaluation of the adsorption properties of the composite towards the dye in the presence of surfactants is an extremely important part of the research because such compounds are present in dyeing baths and can also be released into wastewater. As presented above, they show both an increase and decrease in sorption capacity with respect to the adsorbate. Our previously published research [9] demonstrates that the structure of cationic dye molecules plays an important role in their retention by the composite.

### 2.4. Electrokinetic Experiments

**Figure 6:**
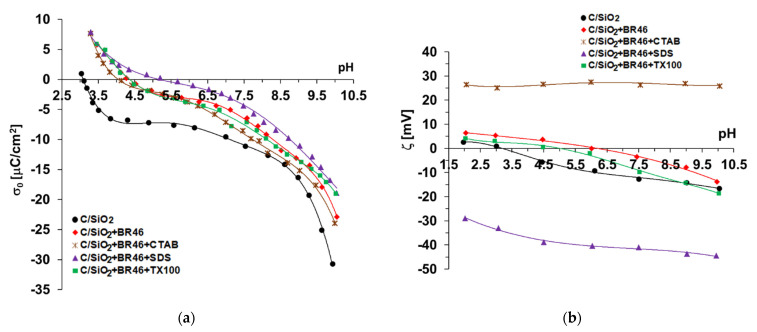
(**a**) Surface charge density versus solution pH and (**b**) zeta potential versus solution pH in the 100 mg/L BR46 + C/SiO_2_—surfactants (0.25 g/L) systems.

The pH changes of the solid surface charge density (*σ*_0_) and the zeta potential (*ζ*) of composite particles dispersed in the dye and surfactant suspensions are presented in Figure 6.

As can be seen in Figure 6a, adsorption of the alkaline dye causes an increase in the surface charge density of the carbon–silica composite in the whole examined pH range and a shift of the pH_pzc_ point (pzc—point of zero charge) towards higher pH values, i.e., from pH 3.1 (system without adsorbates) to pH 4.25 in the presence of BR46. On the other hand, the isoelectric point (iep) for the C/SiO_2_ suspension without additives occurs at a pH of about 3.3 (Figure 6b), which indicates that its value is consistent with the pH_pzc_ value. Under conditions corresponding with the point of zero charge and isoelectric point, the solid particles are characterized by zero surface charge and zero zeta potential, respectively. In the case of point of zero charge, the concentrations of positive and negative charges accumulated in the surface layer are the same. In turn, for the isoelectric point, the concentrations of positively and negatively charged groups located in the slipping plane area within electrical double layer (edl) are identical. The introduction of ionic dye to the suspension results in an increase in the electrokinetic potential (Figure 6b) and the shift pH_iep_ points towards higher pH values, i.e., to the value of 6.0 for BR46.

In the case of the adsorption of relatively large dye molecules, the positive charges present in their structure are most often found in the by-surface layer of the solution. The by-surface layer of the solution is a layer directly adjacent to the solid surface, including the compact part of the electrical double layer that is stationary and rigidly bonded with the solid surface. This leads to an increase in the σ_0_ value. In addition to electrostatic attraction between cationic RB46 dye molecules and negatively charged composite surfaces, hydrogen bonds can be formed. Solid hydroxyl groups (-OH) with amphoteric character form on its surface as a result of the hydroxylation process in aqueous solution. The positively charged groups (-OH_2_^+^) are formed mainly in acidic environments via the attachment of protons to the neutral hydroxyl surface groups. In turn, the negatively charged groups (-O^−^) are created predominantly in basic solutions as a result of the disconnection of protons from the neutral -OH groups. The sum of positively and negatively charged surface groups determines the sign and magnitude of the solid surface charge at a specified solution pH. Solid hydroxyl groups (-OH, -OH_2_^+^ and -O^−^) and nitrogen atoms in the dye molecules participate in this process [40,41]. The latter phenomenon leads to the exposure of the positive charges of the adsorbed dye molecules towards the bulk phase of the solution, which results in the observed increase in the surface charge density. The main phenomena responsible for the obtained changes in the zeta potential are the increase in the number of positive charges in the slipping plane area, as well as its shift from the solid surface, due to the adsorption of large cationic dye molecules [42]. Such a structure of adsorption layers causes a decrease in the aggregate sizes formed in the solution (more effective repulsion between solid particles covered with dye adsorption layers), as presented in Table 4.

In the mixed systems of adsorbates (dye + surfactant), noticeable changes in the course of the σ_0_ and ζ dependencies as a function of the solution pH were observed. In the presence of anionic SDS, there is a further increase in the surface charge density of the solid (compared to suspension containing dye alone). In this situation, dye–SDS complexes are formed, which can adsorb to the surface of the solid. The negatively charged heads of anionic surfactants mainly interact with the positive charges of the dye molecules. Thanks to such a structure, complexes are bound to active sites on the composite surface mainly through hydrogen bonds (using nitrogen atoms of the dye molecules for this purpose). As a result, the SDS carbon tails orient towards the solution, which makes it possible to form a second anionic surfactant adsorption layer with negative heads facing the liquid phase. Thus, the adsorption of the subsequent dye layer is possible (a BR46 adsorption increase in the SDS presence is observed, as shown in Figure 5). The formed RB46 + SDS multilayer causes a considerable shift of the slipping plane towards the bulk solution, which leads to the significant decrease in the zeta potential of such a suspension.

The presence of cationic CTAB and non-ionic TX100 has a noticeable effect on the surface charge density of the carbon–silica composite. This behavior is likely due to the competitive adsorption of these surfactants and dye molecules. The addition of a non-ionic surfactant has a minimal effect on the σ_0_ values in the presence of a dye, whereas the addition of a cationic surfactant causes a noticeable decrease in the σ_0_ value over the pH range above the pzc (i.e., 4.25). This proves the effective adsorption of CTAB molecules in the mixed surface layer, which leads to the creation of an additional number of negatively charged surface groups [43]. At the same time, the adsorption of cationic dye molecules decreases in the CTAB presence. Moreover, adsorptive multilayers of CTAB molecules with positive charges located in the slipping plane area can be formed, as evidenced by a significant increase in the zeta potential of the C/SiO_2_ + BR46 + CTAB system. The possibility of the formation of dye–TX100 complexes through hydrophilic–hydrophobic interactions is very likely [44]. Their binding with the composite surface and multilayer formation considerably increases the amount of adsorbed RB46.

The formation of dye–surfactant complexes was confirmed by the determined sizes of formed aggregates by the solid particles covered with the mixed adsorbates layers (Table 4). This parameter increased in the presence of all examined surfactants, but in the case of CTAB, the largest aggregates were formed. This confirms the proposed adsorption mechanism in the presence of a cationic surfactant, based, inter alia, on the creation of adsorption multilayers. In many cases, absolute zeta potential values exceeding 30 mV (regardless of the sign of the charge) guarantee the effective stabilization of the colloidal suspension [45], which prevents the effective separation of the solid phase with adsorbed substances from the aqueous solution. Therefore, analyzing the obtained electrokinetic data, it can be assumed that the most stable composite + dye + surfactant systems are formed in the presence of cationic CTAB. In turn, C/SiO_2_ + dye suspension containing nonionic TX100 is characterized by the lowest absolute values of electrokinetic potential, which translates into their low stability and the easy separation of the solid from the liquid phase.

## 3. Materials and Methods

### 3.1. Chemicals

**Figure 7:**
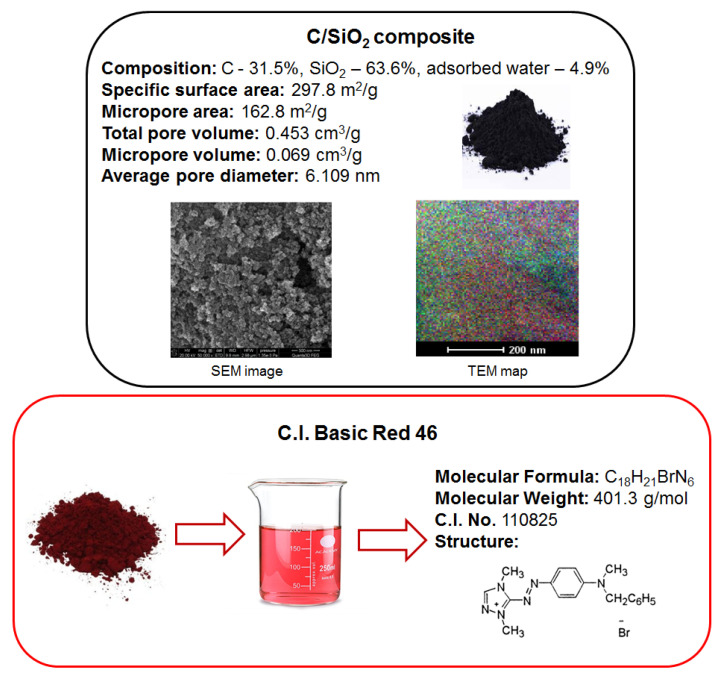
C.I. Basic Red 46 properties.

The following were used as substrates for the synthesis of the composite: pyrogenic silica A-300 (pilot plant of Institute of Surface Chemistry NAN of Ukraine, Kalush, Ukraine) and phenol-formaldehyde resin of novolac type (JSC ‘Ukrainian resins’). The substrates were mixed in a 1:1 weight ratio and grinded by a porcelain ball mill for 2 h. The next step was their pyrolysis for 2 h in argon flow at 1073 K. The adsorbent was thoroughly characterized in terms of composition and structure using appropriate methods and analytical techniques, as described in [9,46]. Analysis of the microscopic images (with a scanning electron microscope (SEM) QuantaTM 3D FEG (FEI Company, Hillsboro, OR, USA) and transmission electron microscope (TEM) TecnaiTM G2 20 (FEI Company)) revealed that the composite had a disordered structure in which the individual components were randomly distributed. A summary of the physicochemical properties of the C/SiO_2_ composite is shown in Figure 7.

Basic single azo type dye, i.e., C.I. Basic Red 46 was used as an adsorbate and was purchased from Boruta-Zachem (Zgierz, Poland). The physicochemical properties of the dye are presented in Figure 7. It is used to dye acrylic fibers and direct printing. The stock dye solution of the initial concentration *C*_0_ = 1000 mg/L was prepared in distilled water, and the working solutions of the defined concentrations were obtained by dilution using volumetric flasks.

Surface-active agents of laboratory grade such as anionic sodium dodecyl sulfate (SDS), non-ionic 2-[4-(2,4,4-trimethylpentan-2-yl)phenoxy]ethanol (TX100) and cationic hexadecyltrimethylammonium bromide (CTAB) were obtained from Sigma-Aldrich (Germany).

The pH values of the suspensions for electrokinetic measurements without and in the presence of dye and surfactants as well as dye solutions for adsorption experiments were determined using HCl and NaOH solutions (POCh Gliwice) with concentrations varying from 0.01 mol/L to 1 mol/L.

### 3.2. Adsorption Tests

In the batch adsorption method, the influence of parameters such as solution pH, BR46 concentration, phase contact time and auxiliaries’ presence (surfactant: SDS, TX100, CTAB) were investigated as factors governing the dye uptake. The adsorption tests were performed at 293, 313 and 333 K using a laboratory shaker Elpin 358+ (Lubawa, Poland) at rotary r = 180 cpm, amplitude A = 8 and at the natural pH of dye solutions (i.e., pH ≈ 4.8). In preliminary adsorption tests, the optimal volume of the solution (0.02 L) and the mass of the adsorbent (0.02 g) were established. The adsorption time varied from 1 to 240 min (kinetic experiments) or was equal to 24 h (equilibrium experiments). The dye concentration was selected in such a way as to reflect the dye content in wastewater. The adsorption experiments were performed in triplicate with reproducibility ± 5%. The conditions for the adsorption experiments are summarized as follows:

- Effect of pH: C_0_ = 100 mg/L of BR46, m = 0.02 ± 0.0002 g, V = 0.02 L, r = 180 cpm, A = 8, T = 293 K, t = 1 h;
- The equilibrium studies: C_0_ = 1, 3, 5, 7, 10, 15,20, 25, 30, 40, 50, 75, 100, 200, 300, 400 and 500 mg/L of BR46, m = 0.02 ± 0.0002 g, V = 0.02 L, r = 180 cpm, A = 8, T = 293–333 K, t = 24 h;
- The kinetic studies: t = 1–240 min, m = 0.02 ± 0.0002 g, C_0_ = 25, 50, 100 mg/L, V = 0.02 L, r = 180 rpm, A = 8, T = 293 K;
- Effect of auxiliaries: C = 0.25 g/L of SDS, TX100 and CTAB; C_0_ = 100 mg/L of BR46, t = 1 h, pH = 4.8 (in SDS presence), pH = 4.4 (in CTAB or TX100 presence).

The composite was separated from the solution via filtration, and then, the solution was analyzed spectrophotometrically (Cary 60 Agilent, Santa Clara, CA, USA) to determine the dye content after the sorption process at the maximum absorbance wavelength.

To validate the best-fitting isotherm model to experimental data, four statistic error functions, namely determination coefficient (*R*^2^), residual sum of squares error (*ERRSQ*/*SSE*), Chi-square (*χ*^2^) and sum of squares regression (*SSR*) were assessed based on the following Equations (11)–(14) [28,46]:

(11) $$R^{2}=1-\frac{\sum_{n=1}^{n}{\left(q_{e,exp}-q_{e,cal}\right)}^{2}}{\sum_{n=1}^{n}{\left(q_{e,exp}-\bar{q_{e,exp}}\right)}^{2}}$$

(12) $$ERRSQ/SSE=\overset{n}{\underset{i=1}{\sum }}{\left(q_{e,exp}-q_{e,cal}\right)}^{2}$$

(13) $$\chi^{2}=\overset{n}{\underset{i=1}{\sum }}\frac{{\left(q_{e,cal}-q_{e,exp}\right)}^{2}}{q_{e,exp}}$$

(14) $$SRR=\overset{n}{\underset{i=1}{\sum }}{\left(\frac{(q_{e,cal}-q_{e,exp}}{q_{e,exp}}\right)}^{2}$$

where *n*—number of experimental data points, *q_e,cal_* (mg/g)—theoretically calculated adsorption capacity and *q_e,exp_* (mg/g)—experimental adsorption capacity.

### 3.3. Electrokinetic Tests

The surface charge density (σ_0_) of the composite without additives and in the tested adsorbate systems was determined using the potentiometric titration method. It consists of recording suspension pH changes as a function of the added titrant volume (in this case, sodium base with a concentration of 0.1 mol/L). The σ_0_ values obtained at different pH values of the system were calculated with the computer program “titr_v3” [47]. The examined solution with a volume of 50 mL was introduced into a thermostated Teflon vessel (RE 204 thermostat, Lauda). A set of glass and calomel electrodes (Beckman Instruments) and a pHM 240 pH-meter (Radiometer) were used to control the pH of the system during titration. The titration process was carried out using an automatic Dosimat 765 (Metrohm) micro-burette and a computer. The examined systems were titrated in the 3–11 pH range, and the suspensions were prepared by adding 0.1 g of C/SiO_2_ to 50 mL of appropriate solution.

Measurements of the electrophoretic mobility of the composite particles dispersed in the aqueous phase without additives and in the tested adsorbate systems enabled the determination of their zeta potential (ζ) using Henry equation [48]. The experiments were carried out using the Zetameter Nano ZS (Malvern Instruments, Malvern, UK). The suspensions were prepared by adding 0.02 g of C/SiO_2_ to 200 mL of the specified solution. The systems prepared in this way were sonicated for 3 min. Then, they were divided into several parts, and a specific pH value was set in each of them (in the range from 2 to 10 ± 0.1). For each sample, the measurements were repeated five times.

Using the static light scattering technique, the size of the C/SiO_2_ particles/aggregates dispersed in the aqueous phase (without and with dye and surfactants) was determined (Zetameter Nano ZS, Malvern Instruments).

The electrokinetic studies were performed in the systems containing BR46 with the concentration of 100 mg/L and surfactants with the concentration of 0.25 g/L.

## 4. Conclusions

This paper presented the adsorptive properties of the carbon–silica composite towards toxic azo dye, i.e., C.I. Basic Red 46 from dyeing baths. The adsorption capacities of C/SiO_2_ towards BR46 ranged from 41.90 mg/g to 176.10 mg/g and increased with a temperature rise from 293 K to 333 K. This confirms the endothermic nature of the process in the BR46-C/SiO_2_ system. The Langmuir isotherm model can be applied for the description of equilibrium data better than Freundlich or Dubinin–Radushkevich models. The negative values of the free energy revealed the spontaneous nature of BR46 adsorption on C/SiO_2_. Kinetic batch adsorption experiments confirmed that BR46 uptake by C/SiO_2_ abide by the pseudo-second-order model rather than pseudo-first order or intraparticle diffusion. The BR46 uptake was influenced by surfactants present in solution. The presence of CTAB in solution affected the decrease in the sorption capacity. An increase in the *q_e_* was observed in the systems containing SDS and TX100. The adsorption of the basic dye caused the increase in the surface charge density and zeta potential of the composite particles in the whole examined pH range (compared to the system without adsorbates), and a shift of the pH_pzc_ and pH_iep_ points towards higher pH values. The sizes of formed aggregates by the solid particles covered with the mixed adsorbates layers increased in the presence of all examined surfactants. In the case of CTAB, the largest aggregates were formed. Apart from the electrostatic attraction between the negatively charged composite surface and the cationic dyes molecules, hydrogen bonds and hydrophobic interactions could occur in the tested suspensions. The addition of surfactants of different ionic character also modified the properties of the solid–solution interface (dye–SDS complex formation, CTAB and BR46 competition and dye–TX100 hydrophobic interactions). This affects not only the mechanism of interaction of dye molecules with the C/SiO_2_ surface, but also the stability of this type of suspension, which is associated with the possibility of effective separation of the solid phase from the liquid medium.

The conducted research may be of great cognitive importance for the development of effective adsorption materials used in technologies for the treatment of textile effluents containing various types of dyes, but additional tests in a column system are required.

## Figures and Tables

**Table 1:** Values of parameters of the Freundlich Langmuir, and Dubinin–Radushkevich isotherms calculated for BR46 adsorption on C/SiO_2_ composite as a function of temperature.

Parameter of Isotherms					
Freundlich		Langmuir		Dubinin–Radushkevich	
T = 293 K					
*k_F_* (mg^1−1/n^ L^1/n^/g)	11.63	*Q_0_* (mg/g)	41.9	*k_DR_* (mol^2^/J^2^)	1.81·10^−7^
1/n	0.362	*k_L_* (L/mg)	0.538	*q_m_* (mg/g)	42.61
*R* ^2^	0.491	*R* ^2^	0.999	*E* (kJ/mol)	1.66
				*R* ^2^	0.986
*ERRSQ*/*SEE*	4940.3	*ERRSQ*/*SEE*	1098.2	*ERRSQ*/*SEE*	148.2
*χ* ^2^	173.1	*χ* ^2^	45.8	*χ* ^2^	4.9
*SSR*	47.6	*SSR*	12.6	*SSR*	0.277
T = 313 K					
*k_F_* (mg^1−1/n^ L^1/n^/g)	18.82	*Q*_0_ (mg/g)	87.31	*k_DR_* (mol^2^/J^2^)	6.72·10^−8^
1/n	0.287	*k_L_* (L/mg)	0.183	*q_m_* (mg/g)	61.43
*R* ^2^	0.47	*R* ^2^	0.996	*E* (kJ/mol)	2.73
				*R* ^2^	0.608
*ERRSQ*/*SEE*	3913.8	*ERRSQ*/*SEE*	5409.5	*ERRSQ*/*SEE*	4082.1
*χ* ^2^	302.2	*χ* ^2^	172.8	*χ* ^2^	242.5
*SSR*	266.8	*SSR*	23.9	*SSR*	205.4
T = 333 K					
*k_F_* (mg^1−1/n^ L^1/n^/g)	18.34	*Q_0_* (mg/g)	176.1	*k_DR_* (mol^2^/J^2^)	1.66·10^−7^
1/n	0.475	*k_L_* (L/mg)	0.11	*q_m_* (mg/g)	117.52
*R* ^2^	0.739	*R* ^2^	0.99	*E* (kJ/mol)	1.73
				*R* ^2^	0.941
*ERRSQ*/*SEE*	22,968.7	*ERRSQ*/*SEE*	5105.7	*ERRSQ*/*SEE*	11,145.2
*χ* ^2^	241.9	*χ* ^2^	106.8	*χ* ^2^	106.9
*SSR*	55.6	*SSR*	6.8	*SSR*	1.5

**Table 2:** Comparison of adsorption capacities of various adsorbents for BR46 [1,2,25,26,27,29,30,31,32].

Sorbent	Equilibrium Results	Ref.
Algerian natural phosphates	*q_e_* = 28.5 mg/g pH = 8, a.d. = 3 g/L	[25]
Biochar from *Chrysanthemum* *morifolium* Ramat straw	*q_e_* = 32.3 mg/g pH = 10, a.d. = 0.02 g/20 mL	[1]
Gypsum	*q_e_* = 39.17 mg/g pH = 8, a.d. = 1 g/L	[26]
Moroccan clay	*q_e_* = 54 mg/g pH = 8, a.d. = 1 g/L	[27]
Ce-doped TiO_2_ nanoparticles loaded on activated carbon	*q_e_* = 58.61 mg/g pH = 5.5, a.d. = 25 mg/20 mL	[29]
Boron waste	*q_e_* = 74.7 mg/g pH = 9, a.d. = 0.1 g/50 mL	[30]
Nickel oxide nanoparticle-modified diatomite (NONMD)	*q_e_* = 105 mg/g pH = 8, a.d. = 0.005 g/25 mL	[2]
Palm bio-waste-derived activated carbon	*q_e_* = 263.16 mg/g pH = 5.5, a.d. = 10 mg/50 mL	[31]
Graphene oxide	*q_e_* = 370.4 mg/g pH = 11, a.d. = 0.4 g/L	[32]
C/SiO_2_ composite	*q_e_* = 41.9–176.1 mg/g pH = 4.7, a.d. = 0.02 g/20 mL	This study

a.d.—adsorbent dose.

**Table 3:** Kinetic parameters calculated from the PFO, PSO and IP models for BR46 sorption from 25–100 mg/L solutions on C/SiO_2_.

Model	Parameter	Initial BR46 Concentration *C*_0_ (mg/L)		
		25	50	100
PFO	*q_e_* (mg/g)	6.1	15.5	36.0
	*k*_1_ (1/min)	0.010	0.012	0.022
	*R* ^2^	0.863	0.984	0.956
PSO	*q_e_* (mg/g)	24.1	32.2	59.2
	*k*_2_ (g/mg min)	0.020	0.006	0.003
	*R* ^2^	0.999	0.996	0.997
IPD	*k_i_*_1_ (mg/g min^0.5^)	5.3	6.1	13.9
	*R* ^2^_1_	0.953	0.999	0.999
	*k_i_*_2_ (mg/g min^0.5^)	0.336	0.998	2.1
	*R* ^2^_2_	0.842	0.978	0.725
	*k_i_*_3_ (mg/g min^0.5^)	0.215	1.0	1.6
	*R* ^2^_3_	0.989	0.995	0.953
*q_e,exp_* (mg/g)		25.0	34.5	60.0

**Table 4:** The size of C/SiO_2_ aggregates formed in the solutions without and with dye and surfactants.

System	Mean Diameter of Aggregates (nm)
C/SiO_2_	790
C/SiO_2_ + BR46	610
C/SiO_2_ + BR46 + CTAB	1080
C/SiO_2_ + BR46 + SDS	940
C/SiO_2_ + BR46 + TX100	860
